# Quantification of Al_2_O_3_ nanoparticles in human cell lines applying inductively coupled plasma mass spectrometry (neb-ICP-MS, LA-ICP-MS) and flow cytometry-based methods

**DOI:** 10.1007/s11051-014-2592-y

**Published:** 2014-08-08

**Authors:** Steffi Böhme, Hans-Joachim Stärk, Tobias Meißner, Armin Springer, Thorsten Reemtsma, Dana Kühnel, Wibke Busch

**Affiliations:** 1 Department of Bioanalytical Ecotoxicology, Helmholtz Centre for Environmental Research - UFZ, Permoserstr. 15, 04318 Leipzig, Germany; 2 Department of Analytical Chemistry, Helmholtz Centre for Environmental Research - UFZ, Permoserstr. 15, 04318 Leipzig, Germany; 3 Department of Powder and Suspension Characterization, Fraunhofer Institute for Ceramic Technologies and Systems - IKTS, Winterbergstr. 28, 01277 Dresden, Germany; 4 Centre for Translational Bone, Joint and Soft Tissue Research, University Hospital Carl Gustav Carus, Technische Universität Dresden, Fetscherstr. 74, 01307 Dresden, Germany

**Keywords:** Inductively coupled plasma mass spectrometry (ICP-MS), Flow cytometry, Size dependency, Cellular internalization, Aluminum oxide

## Abstract

In order to quantify and compare the uptake of aluminum oxide nanoparticles of three different sizes into two human cell lines (skin keratinocytes (HaCaT) and lung epithelial cells (A549)), three analytical methods were applied: digestion followed by nebulization inductively coupled plasma mass spectrometry (neb-ICP-MS), direct laser ablation ICP-MS (LA-ICP-MS), and flow cytometry. Light and electron microscopy revealed an accumulation and agglomeration of all particle types within the cell cytoplasm, whereas no particles were detected in the cell nuclei. The internalized Al_2_O_3_ particles exerted no toxicity in the two cell lines after 24 h of exposure. The smallest particles with a primary particle size (*x*_BET_) of 14 nm (Alu1) showed the lowest sedimentation velocity within the cell culture media, but were calculated to have settled completely after 20 h. Alu2 (*x*_BET_ = 111 nm) and Alu3 (*x*_BET_ = 750 nm) were calculated to reach the cell surface after 7 h and 3 min, respectively. The internal concentrations determined with the different methods lay in a comparable range of 2–8 µg Al_2_O_3_/cm^2^ cell layer, indicating the suitability of all methods to quantify the nanoparticle uptake. Nevertheless, particle size limitations of analytical methods using optical devices were demonstrated for LA-ICP-MS and flow cytometry. Furthermore, the consideration and comparison of particle properties as parameters for particle internalization revealed the particle size and the exposure concentration as determining factors for particle uptake.

**Electronic supplementary material:**

The online version of this article (doi:10.1007/s11051-014-2592-y) contains supplementary material, which is available to authorized users.

## Introduction

The toxic potential of engineered nanoparticles toward human cell lines has been the subject of several in vitro studies (e.g., Bastian et al. (2009); Kühnel et al. (2012); Limbach et al. (2005); Simon-Deckers et al. (2008)). Besides the characterization of the toxic impact, these studies also documented and discussed the role of the internalization of particles into the cells. Techniques such as scanning electron microscopy (SEM) coupled with energy-dispersive X-ray spectroscopy (EDX) allow the determination of both the intracellular localization and the chemical identification of the nanomaterial but yield solely qualitative information (Simon-Deckers et al. 2008; Busch et al. 2011). It has been shown that particles are primarily located in the cytoplasm, entrapped in vesicles or vacuoles (Simon-Deckers et al. 2008), or accumulated in mitochondria and lysosomes. Furthermore, the formation of perinuclear rings by internalized particles was described in several studies (e.g., Zucker et al. (2010) and Busch et al. (2011)). Especially in the fields of medicine and pharmacology, fluorescent or magnetic nanoparticles are used to allow an in vivo detection and direct quantification of internalized particles (Salata 2004). In line with that, the quantification of intracellular nanomaterials became an issue of high relevance for toxicological perspectives as well as for medical applications.

The cellular uptake processes are as diverse as the chemical and physical properties of nanoparticles. Most studies describe a time and concentration-dependent particle internalization, independent of the examined type of nanomaterial (e.g., Radziun et al. (2011); Zucker et al. (2010); Chithrani et al. (2006)). In general, passive diffusion is possible for particles and ions which are smaller than 60 nm. Larger particles and particle agglomerates are internalized by various active phagocytotic or endocytotic mechanisms (Unfried et al. 2007). Jiang et al. (2008) identified particles of a middle-size range (~50 nm) as preferentially taken up, compared to other size ranges. Besides the size of nanoparticles, additional chemical and physical characteristics, such as their surface area (Brown et al. 2001; Hussain et al. 2009), surface coating (Zhang et al. 2002), charge (Gratton et al. 2008), and shape (Chithrani et al. 2006; Gratton et al. 2008), are relevant for uptake and toxicity. These particle properties can be correlated to the appearance of biological effects or the selective accumulation of particles in the cells (Oberdörster et al. 2005; Stoeger et al. 2006; Wittmaack 2007; Waters et al. 2009). The correlation between specific nanoparticle properties and the quantitative amount of internalize nanomaterial were investigated in detail by Au et al. (2009); Chithrani et al. (2006); Limbach et al. (2005) and Zhu et al. (2013). For this goal, they applied inductively coupled plasma mass spectrometry (ICP-MS)-based approaches as well as electron microscopy.

Nebulization-ICP-MS (neb-ICP-MS) is an established and precise method with a detection limit in the ng/l concentration range. Additionally, new applications of ICP-MS allow to gain information about the size distribution of particles (single-particle ICP-MS, e.g., Mitrano et al. (2012)) and the local distribution of particles (laser ablation ICP-MS, e.g., Drescher et al. (2012)). The laser ablation inductively coupled plasma mass spectrometry (LA-ICP-MS) system is an established method to investigate the element composition of geological and archeological samples (Russo et al. 2002). Furthermore, the applicability as a visualization method with a high spatial resolution has been described (Becker et al. 2007; Wang et al. 2013).

Another way to determine the internalization of nanoparticles into cells is the indirect measurement of the cellular granularity by flow cytometry methods (Palecanda and Kobzik 2000). As the incorporation of nanoparticles changes the granularity of living cells, the detection of the side scattered light in a flow cytometer can be used as an optical detection method to study nanoparticle uptake (Zucker et al. 2010; Busch et al. 2011). It is, however, of high interest whether the indirect flow cytometry measurements can be correlated directly to internal particle concentrations. In order to observe the behavior, destination, and distribution of nanomaterials in biological tissues, especially in cancer cells, flow cytometry has been applied in medical research (Suzuki et al. 2007).

In this study, we investigated the quantitative uptake of three differentially sized Al_2_O_3_ particles in order to compare three different quantification methods. Al_2_O_3_ particles were chosen because they are not toxic to human cells (Radziun et al. 2011), and they are commercially available in different sizes. In the first step, we investigated the uptake of the particles into the cells, and the distribution within cells qualitatively applying electron microscopy. The chemical and physical properties, the stability, and the behavior of the particles in the relevant media were characterized in detail, and the sedimentation velocity was calculated in order to determine the influence of a restricted bioavailability of nanomaterial to the cell monolayer. Second, three independent analytical methods (digestion combined with neb-ICP-MS, LA-ICP-MS, and flow cytometry) were used to determine the internal particle concentrations within the cell lines. The results were compared by normalizing the mass of the internalized aluminum oxide to the respective cell surface area. Finally, the relationship between the intracellular concentrations and different particle properties was assessed by a direct comparison of different particle characteristics.

## Materials and methods

### Initial Al_2_O_3_ particle characteristics

The three Al_2_O_3_ particles were purchased as powders from different manufacturers. They are named in the following as Alu1 (AEROXIDE^®^ Alu C, Evonik Degussa GmbH), Alu2 (TAIMICRON^®^, TM-DAR, Taimei Chemicals Co., LTD.), and Alu3 (NABALOX^®^, NO-625-10, Nabaltec AG). The morphology of the particle powders was investigated by scanning electron microscopy (SEM, Zeiss Leo 982 FEG, Carl Zeiss SMT AG). The N_2_-BET-specific surface area was obtained by gas adsorption measurement according to the Brunauer-Emmet-Teller method (ASAP 2010 Analyzer, Micromeritics GmbH), and the crystalline structure of the powders was determined by X-ray diffraction (XRD7, Seifert-FPM). The powder density was determined using helium pycnometry (Penta Pycnometer, Quantachrome GmbH & Co. KG). Theoretical primary particle size (x_BET_) was calculated on the basis of the BET and density values obtained.

### Preparation and characterization of particle suspensions

Particle suspensions in cell culture media were produced according to Meißner et al. (2010). Briefly, the stock suspensions of particles (500 mg/l) were prepared in pure water under addition of 0.05 % (w/v) sodium hexametaphosphate (SHMP, Merck KGaA) as stabilizing agent. SHMP is non-toxic in this concentration range and causes electrostatic repulsion between the particles. The suspensions were sonicated with an ultrasonic disintegrator equipped with a 14-mm sonotrode (UDS 751, Topas GmbH) for de-agglomeration. The particle size distribution and agglomeration behavior of the smallest (Alu1) and middle-sized particles (Alu2) in suspension were investigated by dynamic light scattering (DLS, Zetasizer Nano ZS, Malvern Instruments Ltd.), and the polydispersity index was determined. For the larger particles (Alu3), laser light diffraction (Mastersizer 2000, Malvern Instruments Ltd.) was applied to measure the particle size in suspension, whereby different quantiles of the volume-weighted distribution help to gain information about the particle size distribution. The zeta potential of the stock suspensions was determined by measuring the electrophoretic mobility of the particles by means of electrophoresis. The Smoluchowski equation was taken to convert the electrophoretic mobility into the zeta potential. Stock suspensions were stored at 4 °C for a maximum of eight weeks. In order to estimate the influence of the sedimentation rate of the particles to the cell uptake, the sedimentation velocity (v_p_) was calculated for a spherical particle shape with the determined hydrodynamic particle diameters for a temperature of 37 °C according to Stoke´s law:

$$v_{P} = \frac{{2r^{2} g*(\rho _{P} - \rho _{f} )}}{{9*\eta }},$$

where r is the radius of the particle, g is the gravitational acceleration, η is the dynamic viscosity, and ρ_p_ and ρ_f_ describe the density of the particle and the surrounding media, respectively.

### HaCaT and A549 cell culture

Two different human cell lines were used, representing model cell lines for an inhalation-related (human lung epithelial cells, A549) and a dermal-related uptake pathway (human skin keratinocytes, HaCaT, Boukamp et al. (1988)) both with a cell diameter of approximately 10 µm. Both cell lines were cultivated in RPMI medium (RPMI 1640, Biochrom AG) supplemented with 10 % fetal bovine serum (FBS, (v/v), Sigma Aldrich) in 75 cm^2^ flasks at 37 °C in a humidified and 5 % CO_2_ atmosphere. A sub-cultivation was done every 3–4 days. The cell monolayer was washed two times with Versene (0.48 mM EDTA/PBS buffer solution, Gibco^®^, Invitrogen), detached with trypsine (0.25 % (v/v) in phosphate-buffered saline (PBS)), and the cells were counted with a hemocytometer.

### Exposure of cells

Cells were seeded in different densities, depending on the plate format used in the actual experiment (Table 1). By microscopic inspections, it was guaranteed that subsequent experiments were performed exclusively on complete closed cell monolayers. Independent of the exposure setup, the medium height for all experiment was 2 mm. Before usage, particle stock suspensions were re-dispersed by shaking and sonication in an ultrasound bath (Eurolab USR 30H, Merck KGaA) for 5 min before they were diluted in cell culture media and subsequently applied to the cells. For all experiments, concentrations of 10, 20, 30, 40, and 50 mg/l were used. By dividing the total offered particle amount through the cell surface area, the exposure was expressed as µg Al_2_O_3_/cm^2^ cell layer in the following. After 24 h of exposure, cells were washed twice with Versene to remove particles loosely bound to the cell surface, which was controlled by light microscopy. Controls of water and water with 0.05 % SHMP were included in all experiments. All results represent a minimum of three biological replicates.

**Table 1:** Exposure designs for the cell viability tests and the different exposure experiments

Experiment	Plate format	Cell density (cells/ml)	Exposure volume (ml)
Cell viability test	24-well plates, 1.9 cm^2^	5 × 10^4^	1
Flow cytometry	6-well plates, 9.6 cm^2^	20 × 10^4^	2
Neb-ICP MS	6-well plates, 9.6 cm^2^	20 × 10^4^	2
LA-ICP MS	glass slides, 12.95 cm^2^	17 × 10^4^	3

### Analysis of cellular particle uptake by light and electron microscopy

The particle distribution within the cells was investigated after 24 h of exposure to the highest concentration of 50 mg/l. Particle-treated cells were observed by an inverse microscope (Leica DMI 4000B, magnification 200×) connected to a Leica DFC 290 HD camera. Detailed analyses of the localization of particles inside the cells were performed by scanning electron microscopy (SEM, Philips ESEM XL 30 FEG) coupled with energy-dispersive X-ray spectroscopy (EDX with EDAX detecting unit, EDAX Inc.) for element analysis. Therefore, cell pellets containing ~10 × 10^5^ cells were fixated with 2.5 % glutaraldehyde in PBS after exposure. After washing with PBS, the samples were stained with osmium tetroxide, washed with deionized water, dehydrated with a graduated acetone series, including an additional staining step with uranyl acetate, and finally embedded in epoxy resin. In the last step, samples were cut by a microtome (Leica EM UC6, Leica Microsystems GmbH) and measured by SEM-coupled EDX to determine the element distribution (Bastian et al. 2009; Kühnel et al. 2012).

### ICP-MS analysis

Two approaches for the ICP-MS-based quantification of particle uptake into human cells were developed. First, an acid digestion was followed by analysis with a standard neb-ICP-MS measurement, and second, a laser ablation system was coupled with ICP-MS (ELAN DRC II, Perkin Elmer Sclex.) to analyze thin films or cell monolayers. For both experimental setups, cells were exposed for 24 h to all types of particles in a concentration range between 10 and 50 mg/l and washed twice with PBS to remove particles loosely bound to the cell surface. Scandium (standard solution 1 g/l, Merck KGaA) with a concentration of 10 µg/l was measured as an internal standard element for all samples. Raw aluminum data of ICP-MS measurements were corrected by the multiplication with the Al_2_O_3_/Al mass ratio factor of 1.89. Finally, all received results of the neb-ICP-MS and LA-ICP-MS experiments were extrapolated to the amount of particles per cm^2^ cell layer.

### Digestion for nebulization-ICP-MS

The complete destruction of cells and dissolution of aluminum oxide nanoparticles were achieved by acid digestion. After the exposure period, cells were washed, detached with trypsine, and centrifuged. The cell pellet was stored at −20 °C and re-suspended in water for the digestion process. An acid digestion method with addition of 10 ml hydrochloric acid (30 %, suprapur, Merck KGaA) and 500 mg potassium chlorate (for analysis, Merck KGaA) in separate reaction vessels inside the digestion system (HPA-S, AntonPaar GmbH) was developed. During the reaction, elemental chlorine was formed at a temperature of 250 °C, a pressure of 100 bar, and a reaction time of 2 h for small Al_2_O_3_ particles (Alu1, Alu2) and 4 h for micron-sized particles (Alu3). These long reaction conditions are necessary to gain the best recovery rates (~99 ± 5 %). After digestion and appropriate dilution, all samples and controls were analyzed by neb-ICP-MS connected to an auto sampler (Perkin Elmer SC-2 DX). An external calibration was performed by analyzing an aluminum standard solution (standard solution 1 g/l, Merck KGaA).

### Laser ablation ICP-MS

The amount of aluminum in cells grown on glass slides was measured by LA-ICP-MS. After exposure, the cell layer was carefully washed several times with PBS and allowed to dry under a laminar flow. An external calibration was performed by application of 30 µm thick agarose gels, according to an earlier protocol (Stärk and Wennrich 2011). The external calibration curve was generated by spiking agarose gel slides with the individual particle type. Gels were prepared by dissolving 100 mg agarose (for electrophoresis, Merck KGaA) in water. After addition of 5 ml ammonium acetate buffer (for analysis, 2 mol/l, Merck KGaA) and scandium as internal standard (scandium standard solution, 1 g/l, Merck KGaA), the gel solution was filled up to 10 ml with water. The gel solution was heated to 90 °C and homogenized by carefully shaking. Before pouring the gel (4 ml of gel solution), a defined particle amount was added to the solution. Gel slides were allowed to dry and were measured by a laser ablation (LSX 500, Nd:YAG, CETAC) coupled with an ICP-MS system. The laser was adjusted to 50 % energy, 200 µm spot size, 100 µm/sec scan rate, and 20 Hz pulse repetition rate. The nebulizer was used in parallel to merge the laser aerosol with a blank solution, guarantying constant wet plasma conditions over all experiments. Therefore, the laser gas stream was laterally connected via a tube to the spray chamber (Scott type rython, Perkin Elmer Sclex.). For an easier evaluation, a line of 5,000 µm length was ablated, leading to a total ablation area of 1 mm^2^ which represents a cell number of ~39,000 cells. A minimum of three lines was ablated for each gel slide. The amount of aluminum measured for each slide was then corrected by a factor of 1.141 according to the concentration gradient at the boundary of the slide (Stärk and Wennrich 2011). Finally, the concentration of aluminum was extrapolated to the whole slide area of 1295.16 mm^2^ and transformed into concentration values of µg Al_2_O_3_/cm^2^ cell layer.

### Flow cytometry

For flow cytometry measurements, cells were exposed for 24 h to particles in a concentration range of 10–50 mg/l, washed twice with PBS, and detached with accutase (PAA Laboratories). Detached cells were washed again in PBS and centrifuged. The cell pellets were re-suspended in PBS, stored on ice, and immediately measured by a flow cytometer (FACS Calibur, Becton–Dickinson). The flow cytometer contains a 488-nm air-cooled argon-ion laser and a FSC diode detector. A flow rate of 35 µl/min was chosen. For each experiment, 10 × 10^4^ viable cells were counted, and the median side scatter (SSC) and forward scatter (FSC) values were measured. The SSC values represent the granularity of the cells (Zucker et al. 2010). The SSC values were converted into the particle concentration per sample using the individual particle suspensions in a concentration range from 2 to 80 mg/l without cell matrix for calibration. Flow cytometry data were analyzed using CellQuest Pro software (Becton–Dickinson) and the FlowJo Software (version 7.2.2; Tree Star Inc.). Finally, all received results were extrapolated to the amount of particles per cm^2^ cell layer. This allows a direct comparison of the results with those achieved by neb-ICP-MS and LA-ICP-MS, independent of the exposure volume, the total cell number, or the exposure format.

## Results

The aim of this study was the appropriate quantification of particle concentrations within human cells, by the application of three different analytical techniques. First, the chemical and physical particle properties were characterized in detail, and the toxic potential of the investigated nanomaterials was determined. The second step comprised the detection of the internalized particles within the cells, prior to a quantification which was the third and major aim of the study.

### Characteristics of aluminum oxide powders and suspensions

**Fig. 1:**
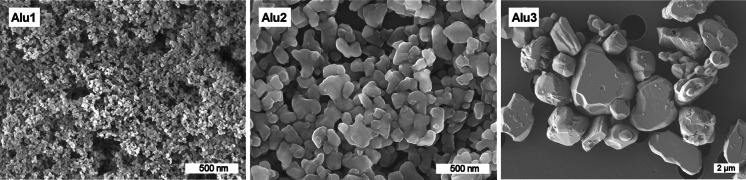
SEM images of aluminum oxide powders (Alu1—AEROXIDE^®^ Alu C, Alu2—TAIMICRON^®^, Alu3—NABALOX^®^)

The parameters determined for the particle powders and suspensions are summarized in Table 2. SEM pictures show a spherical to irregular shape of the particles, with larger agglomerates or aggregates observed for the small (Alu1) and middle-sized material (Alu2) (Fig. 1). The particle surface area (BET) for the particle powders ranges between 117 m^2^/g (Alu1), 13.5 m^2^/g (Alu2), and 2 m^2^/g (Alu3). Under inclusion of the density (3.6 g/cm^3^ for Alu1, 4.0 g/cm^3^ for Alu2 and Alu3) and the BET values, a primary particle size (x_BET_) of 14 nm (Alu1), 111 nm (Alu2), and 750 nm (Alu3) was calculated. The diameter of the particles in suspension ranges between 127 nm (Alu1) and 2.5 µm (Alu3) (Table 2). The high absolute values of the zeta potential imply strong electrostatic repulsion forces, indicating a stable particle suspension. Fetal bovine serum was supplemented to the medium and is known as an additional particle stabilizing agent under physiological conditions (e.g., Meißner et al. (2010)). However, agglomeration in cell culture media and increasing particle diameters were observed by DLS during the exposure period. The sedimentation velocity after the exposure period was calculated to be 2.5, 7, and 1,270 mm/24 h for Alu1, Alu2, and Alu3, respectively. Considering the media height of 2 mm in the cell culture vessels, the micron-sized (Alu3), middle-sized (Alu2), and smallest (Alu1) particles were expected to be settled completely after 3 min, 7 h, and 20 h, respectively. The micron-sized particles (Alu3) settled most rapidly. For the smallest particles (Alu1), it is assumed that the majority of particles had reached the cell surface during the exposure time of 24 h.

**Table 2:** Chemical and physical characteristics of the investigated aluminum oxide powders and suspensions

	Alu1	Alu2	Alu3
Product name	Alu C	TM-DAR	NO 625-10
Powder characteristics			
Crystal structure	γ-Al_2_O_3_; δ-Al_2_O_3_	α-Al_2_O_3_	α-Al_2_O_3_
BET (m^2^/g)^a^	117	13.5	2
*x*_BET_ (nm)^b^	14	111	750
ρ (g/cm^3^)	3.3	4.0	4.0
Suspension characteristics			
*x* (nm)	127^c^	186^c^	2,500 (*x*_50,3_)^d^
*x*_10,3_; *x*_90,3_	–	–	1,200; 4,900^d^
Polydispersity index	0.19	0.10	–
Zeta potential (mV)	−58	−64	−83

^a^Specific surface area

^b^Primary particle size

^c^Hydrodynamic diameter x_DLS_ (DLS)

^d^Particle size in suspension measured by laser light diffraction

### Particle internalization, distribution, and toxicity to cells

**Fig. 2:**
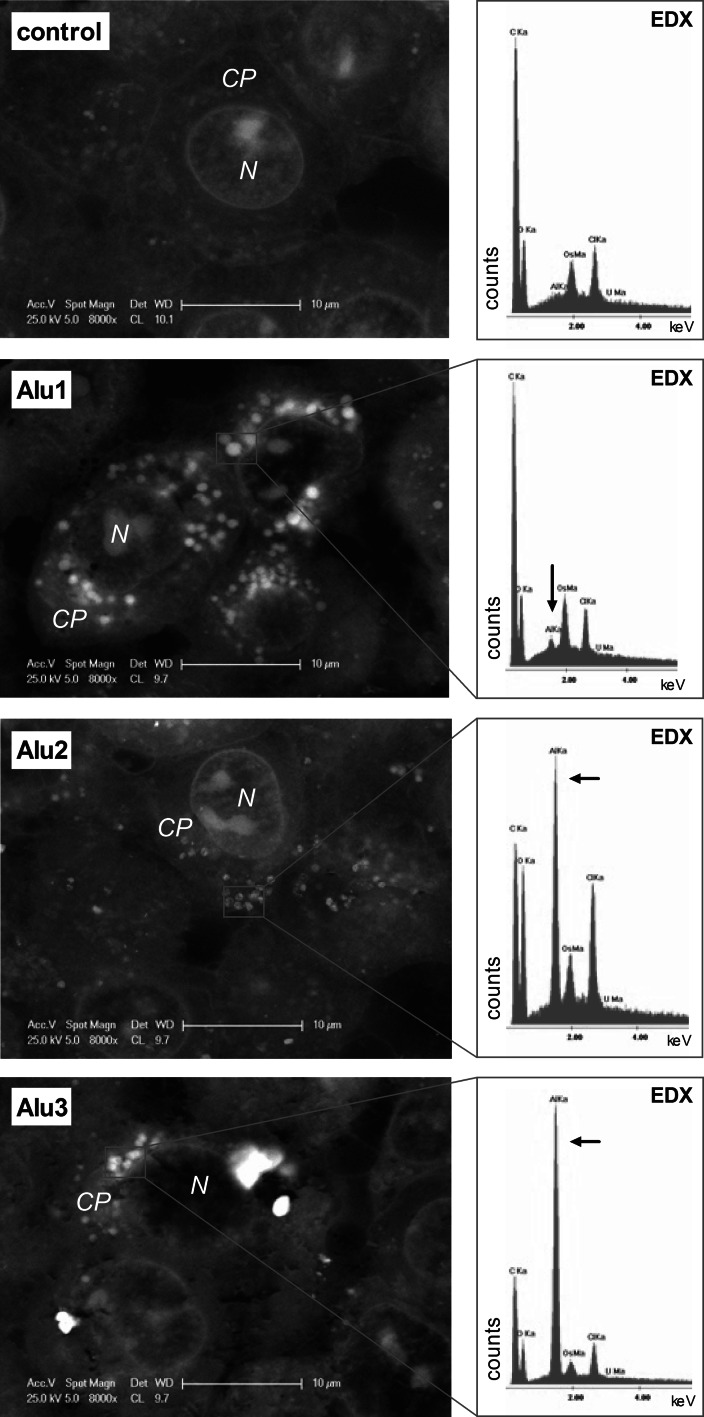
SEM micrographs of sections of embedded human lung epithelial cells (A549) exposed to 10.4 µg Al_2_O_3_/cm^2^ cell layer and medium without particles (control) for 24 h. The area subjected to EDX analysis is marked with a red rectangle. Heavy elements (e.g., aluminum) appear as light electron dense areas. No aluminum was detected in control cells. All three types of particles were incorporated by the cells and were located in the cell cytoplasm (*CP*). No particles were detected in the cell nucleus (*N*)

Observations performed with light microscopy clearly showed that particles became associated to cells upon 24 h of exposure (see Supplemental Material, Fig. S1). For the three particle types investigated, distinct differences of the particle distribution in the cell cytoplasm were observed, with a homogenous distribution of the small particles (Alu1) and a heterogeneous, random localization of the micron-sized particles (Alu3). In contrast, the middle-sized particles (Alu2) exhibit the formation of ring patterns around the cell nuclei, described by Zucker et al. (2010) as perinuclear rings for titanium dioxide particles. To confirm the internalization of particles in cells, SEM coupled with an element detection unit (SEM/EDX) was performed (Fig. 2). In the cytoplasm of both cell lines, electron dense material was detected after the exposure with aluminum oxide particles (the results for the HaCaT cells are provided in the supplemental material (Fig. S2)). No such material was observed in control cells and in the cell nucleus of all treatments. Electron dense material in the cells was identified as aluminum, showed by the presence of an aluminum peak in the spectra measured by EDX, and exemplarily shown for single A549 cells in Fig. 2. The internalized particles formed agglomerates inside the cells.

The toxicity of the different aluminum oxide particles was investigated prior to the measurements of internal concentrations. All details and results are presented in the supplemental material. For this purpose, a combined cell viability assay, using AlamarBlue and CDFA-AM as fluorescence indicator dyes for metabolic activity and membrane integrity, respectively, was performed. For all types of particles and both cell lines, no toxicity was observed, including the highest test concentration of 50 mg/l, which equals 26.3 µg Al_2_O_3_/cm^2^ cell layer (Fig. S3). By the selection of those non-toxic Al_2_O_3_ particle concentrations for the internal concentration measurements, the investigation of vital cells was ensured.

### Quantification of nanoparticle uptake by nebulization-ICP-MS

**Fig. 3:**
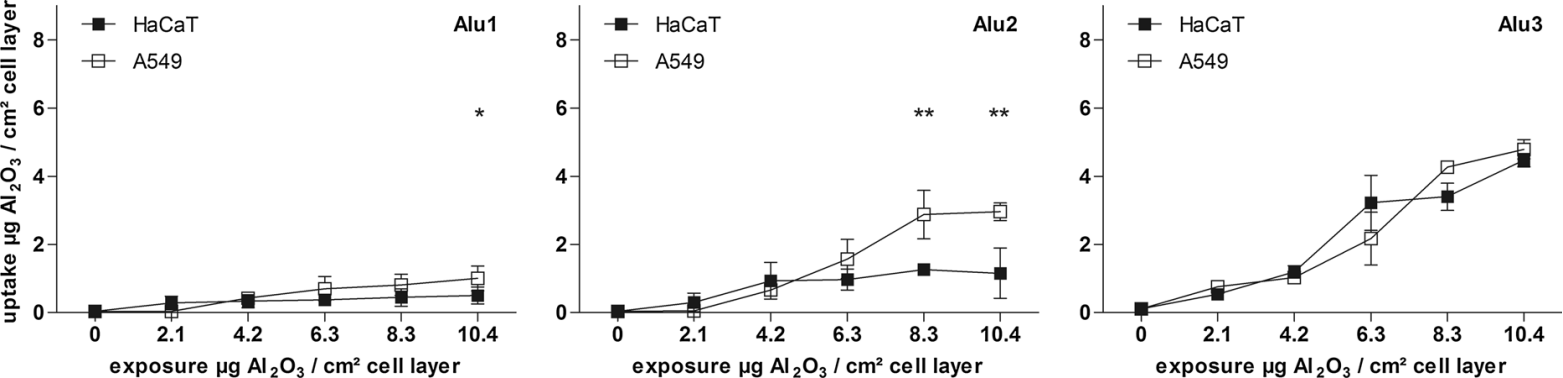
Internal concentrations of Al_2_O_3_-NP over various exposure concentrations as obtained by digestion combined with nebulization-ICP-MS measurements after 24 h of exposure. Results are presented for each particle type and cell line in one diagram. Data are shown as mean ± standard deviation of at least three independent replicates. Significant differences between the cell lines were tested with two-way ANOVA (*p < 0.05, **p < 0.01)

A quantification of the aluminum concentration inside the cells was achieved by an adjusted acid digestion method followed by neb-ICP-MS measurement. After calibration with an aluminum standard solution, the aluminum oxide content was calculated in correspondence to the area of the exposed cell monolayer. In general, a particle type-dependent increase in the uptake concentrations from the smallest (Alu1) to the micron-sized particles (Alu3) was observed (Fig. 3). For example, the highest aluminum oxide amounts were detected for the cells exposed to 10.4 µg Al_2_O_3_/cm^2^ cell layer of micron-sized particles (Alu3). Further, a clear exposure concentration-dependent increase of particle amounts within cells was detected for all treatments. Additionally, the internal concentration of the middle-sized Al_2_O_3_ particles (Alu2) is significantly different between the two cell lines. A twofold higher amount of aluminum oxide was measured in the lung epithelial cells compared to the skin keratinocytes after exposure to 8.3 and 10.4 µg Al_2_O_3_/cm^2^ cell layer, respectively. This is also observed in cells exposed to the smallest particles (Alu1), whereas the difference between the internal concentrations in the two cell types is marginal and only significant for the highest exposure concentration. The relative uptake rates (% uptake) of particle internalizing cells were constant over the tested concentration range and vary between 8 (Alu1), 16–21 (Alu2), and 38–39 % (Alu3) of the totally offered material (Fig. S4).

### Quantification of nanoparticle uptake by LA-ICP-MS

**Fig. 4:**
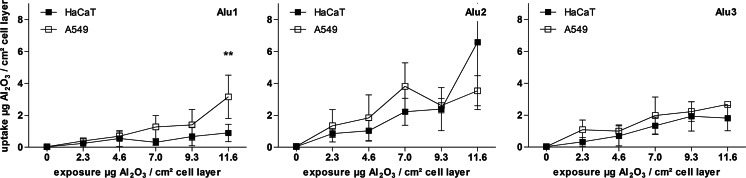
Internal concentrations of Al_2_O_3_-NP over various exposure concentrations as obtained by LA-ICP-MS measurements after 24 h of exposure. Results are presented for each particle type and cell line in one diagram. Data are shown as mean ± standard deviation of at least three independent replicates. The obtained LA-ICP-MS values are converted into the internalized particle mass by external calibration using individually spiked agarose gels. Significant differences between the cell lines were tested with two-way ANOVA (**p < 0.01)

A LA-ICP-MS method was developed to quantify the amount of aluminum within cells grown on glass slides. The experimental setup was designed with the aim to avoid any further sample preparation. Since the laser ablation system is limited due to its optical properties and the quality of the plasma plume, which derives from the laser-sample-ablation process, a particle size correlated effect can be detected with this method, independent of the inserted laser energy. This results from an incomplete destruction of large particles and leads to an underestimation of signal intensities. To compensate for the occurrence of the observed particle size effects, an external calibration was performed by spiking agarose gels with the individual particle suspensions. For all particle exposed cells, a concentration-dependent increase in aluminum oxide concentration was observed (Fig. 4). The highest particle concentrations were measured in the cells exposed to the middle-sized particles (Alu2). A significant difference between the two cell lines was only observed for Alu1, which was taken up to a higher extent by A549 cells. Furthermore, a high variability between the replicates was observed. Regarding the obtained results for the micron-sized particles (Alu3), uptake concentrations lower than 2.5 µg Al_2_O_3_/cm^2^ cell layer were measured. This value is 45 % lower than determined by digestion combined with neb-ICP-MS experiments.

### Quantitative flow cytometry studies

**Fig. 5:**
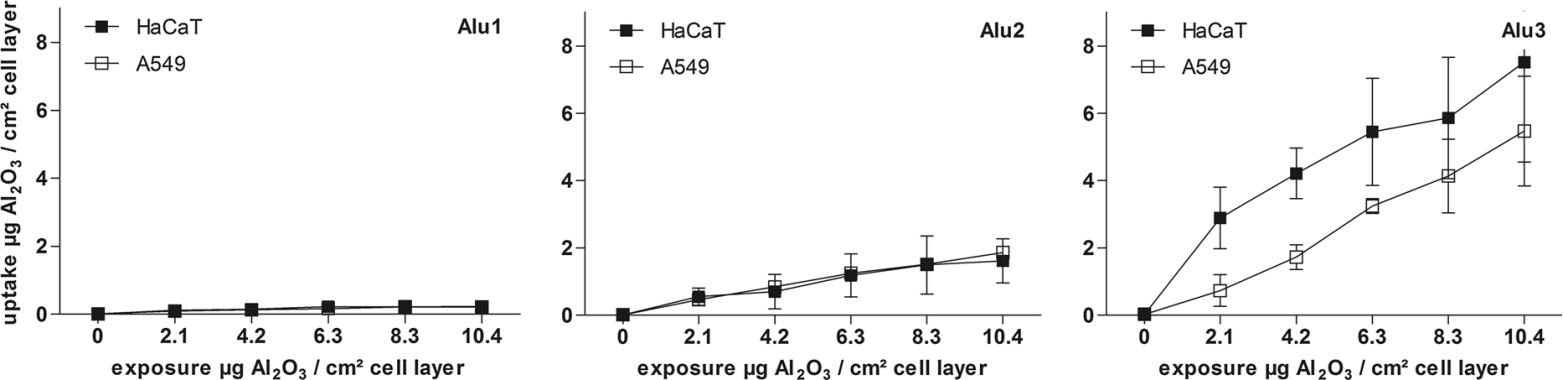
Internal concentrations of Al_2_O_3_-NP over various exposure concentrations as obtained by flow cytometry measurements after 24 h of exposure. Results are presented for each particle type and cell line in one diagram. Data are shown as mean ± standard deviation of at least three independent replicates. The obtained SSC values are converted into the internalized particle mass by external calibration curves. Two-way ANOVA revealed no significant differences between the cell lines

Cells treated with nanoparticles show an increase in granularity and consequently in the SSC-signal intensity, which is measurable by flow cytometry. In the first step, we established calibration curves for the three aluminum oxide particle types in a concentration range from 2 to 80 mg/l, without cells as matrices. A concentration-dependent increase in the SSC value was observed, and a linear correlation between the SSC values and the particle concentrations could be determined between 0 and 50 mg/l for all particle types. With the help of these calibration curves and by the subtraction of the SSC values for the control cells, the obtained SSC values for the exposed cells were converted into uptake concentrations of particles. Only a minor concentration-dependent increase in the signal intensity could be observed for the smallest Al_2_O_3_ particles (Alu1). In contrast, the uptake concentrations detected for the middle (Alu2) and micron-sized particles (Alu3) show a clear concentration dependency (Fig. 5). A high variability between the replicates was observed for the micron-sized particles (Alu3). Compared to the results of neb-ICP-MS, the flow cytometry measurements are based on the unspecific optical detection of changes in the granularity of the cellular system and could not indicate significant differences between the cell lines.

## Discussion

In this study, two human cell lines were exposed to three different types of aluminum oxide particles, with primary particle size medians of 14, 111, and 750 nm, respectively. The observed instability of aluminum oxide particles within cell culture media containing serum albumin by DLS measurements is in accordance with findings of Radziun et al. (2011) and Simon-Deckers et al. (2008) who showed the agglomeration and sedimentation of aluminum oxide nanoparticles in cell culture medium irrespective of the presence of fetal bovine serum. This seems to be an aluminum oxide-specific phenomenon, as serum albumin was described to stabilize, e.g., titanium dioxide or tungsten carbide nanoparticles within the relevant culture media (e.g., Ji et al. (2010) and Meißner et al. (2010)). The instability of aluminum oxide particles in cell culture medium could be due to the positive particle surface charge under physiological conditions. In our experiments, SHMP with negative binding sites was introduced as additional stabilizing agent, what could shield the particle surface by preventing an additional binding of serum albumin. By calculating the sedimentation velocity of all particles, it was estimated that all types of particles had settled completely during the exposure time of 24 h. Especially the micron-sized particles (Alu3) settled extremely rapid with a sedimentation velocity of more than 1 m/24 h. The low sedimentation velocity of 2.5 mm/24 h for the smallest particles (Alu1) could lead to a reduced bioavailability. This has to be considered when interpreting the uptake data.

The internalization and localization of particles in the cell cytoplasm were investigated by light and electron microscopy. Both methods revealed an accumulation and agglomeration of all types of particles in the cell cytoplasm, whereas no particles were found to be located in the cell nuclei. The homogenous distribution of the smallest (Alu1) in contrast to a heterogeneous, random localization of the micron-sized particles (Alu3) in the cell cytoplasm may be due to the size and agglomeration behavior of the particles. Furthermore, in particular, the middle-sized particles (Alu2) formed perinuclear rings, as described previously for polymer, titanium dioxide, and tungsten carbide nanoparticles (Gratton et al. 2008; Zucker et al. 2010; Busch et al. 2011). With this data we show that the same material can behave qualitatively different within cells depending on physical properties such as particle size. We also conclude that this is cell type independent, at least in our study, as the different patterns of particle distribution are similar in lung and skin cells.

Although the particles were internalized into the cells, no acute toxic effects up to a concentration of 50 mg/l and an exposure duration of 24 h were detected. Simon-Deckers et al. (2008) also described non-toxic effects of Alu1 (AEROXIDE^®^ Alu C) toward A549 cells after 72 h of exposure to a maximum concentration of 100 µg/ml. One reason for the low toxicity is the low solubility of aluminum oxide particles, preventing the occurrence of toxic effects caused by dissolved ions which leach out of the particle surface once inside the medium or the cell. However, as an export of particles out of cells has not yet been described, no predictions for long-term or chronic effects can be derived from acute cell toxicity assays.

**Fig. 6:**
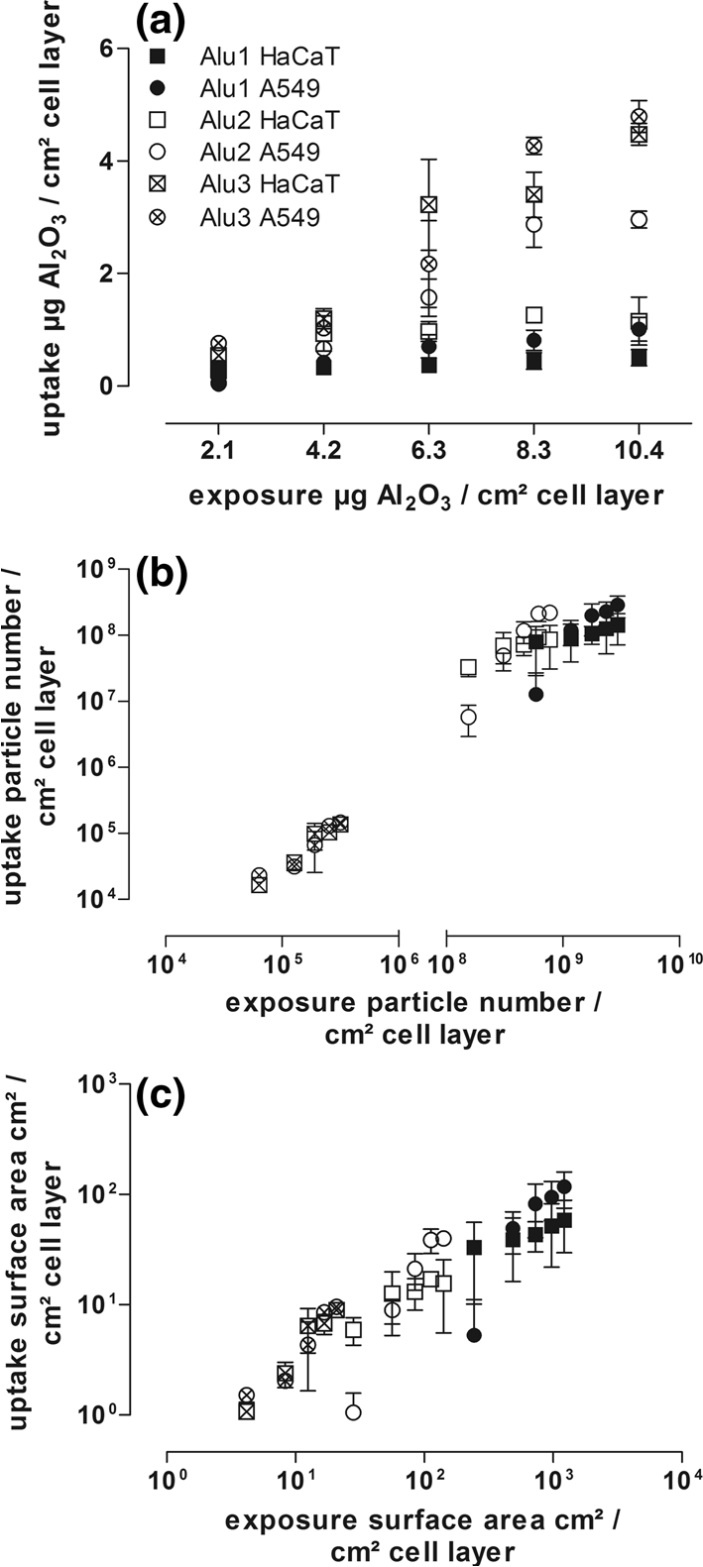
Correlation between the nominal exposure and measured uptake concentrations by digestion combined with neb-ICP-MS expressed by **a** particle mass, **b** particle number calculated with the hydrodynamic particle diameter, and **c** particle surface area. These comparisons included all results for both cell lines after 24 h of exposure for all test concentrations. Data are shown as mean ± standard deviation of at least three independent replicates

The uptake amounts of incorporated particles were quantified by digestion combined with neb-ICP-MS, LA-ICP-MS, and flow cytometry measurements. The results of all three methods confirmed a concentration-dependent particle internalization, with minor significant differences between the lung and the skin cell lines. A concentration-dependent uptake of Al_2_O_3_ particles into murine and human skin fibroblasts was measured previously by Radziun et al. (2011) using ICP-OES. In their study, only one type of a self-synthesized Al_2_O_3_ particle with a median particle size of 50–80 nm and an exposure duration of 24 h was applied. The quantity of uptake reported by Radziun et al. (2011) cannot be compared with our results because they normalized it to the protein content rather than to the cell surface. In our study, additionally, a size dependency with respect to particle internalization was observed (Fig. 6a). Higher aluminum amounts in the cells exposed to larger particles (Alu2/3) were measured. The influence of particle size on the particle uptake may be related to the size and density-dependent sedimentation velocity. Several studies have already discussed the influences of gravitation, diffusion, and agglomeration on the bioavailability of particles to the cell monolayer (Teeguarden et al. 2007; Hinderliter et al. 2010). According to these particokinetic dosimetry considerations, it is necessary to measure the offered material to the cell surface, to calculate the effective dose, which could be magnitudes below the nominal exposure concentration. Principally, large and dense particles with a diameter >100 nm are most affected by gravitation forces, whereas particles below 100 nm are stronger influenced by diffusion processes (Teeguarden et al. 2007). Future studies should elucidate the influence of sedimentation on internal concentrations by extending the exposure times for smaller particles. In general, the cellular internalization of nanoparticles seems not to be limited by the particle size, the quantity of uptake events, or the nominal mass, as no kind of saturation effect with respect to the intracellular Al_2_O_3_ amounts was detected within an exposure duration of 24 h. These observations were in contrast to the findings of Davda and Labhasetwar (2002) who described a higher efficiency of polymer nanoparticle uptake at low exposure concentrations and the achievement of a cell saturation capacity. In this case, the particle composition might have influenced the uptake behavior of the cells. In addition, another cell type was used in their study which could lead to different uptake mechanisms and uptake behavior.

In addition to the size and concentration-dependent internalization, the correlation of the internal aluminum oxide amounts and the applied particle numbers or particle surface area were analyzed (Fig. 6b and 6c). The correlation of the obtained results with particle properties was performed, according to the knowledge of measured particle characteristics, such as particle density and size. Our results indicate that with decreasing particle diameter, a larger number of particles were offered and also internalized. The same is true regarding the particle surface area. Previous studies correlated biological effects to different particle dose metrics (Oberdörster et al. 2005; Waters et al. 2009). Especially, biological responses, such as lung inflammatory responses, have been correlated with the dose metrics of mass concentration, particle number, and particle surface area in order to find the measure that is directly related to effects (Nel et al. 2006). So far, most studies indicate that the particle surface area could be the main influencing factor for biological effects (Oberdörster et al. 2005; Stoeger et al. 2006; Waters et al. 2009). In order to validate this correlation between offered and internalized concentrations, measured as particle surface area, additional experiments are necessary applying same concentrations of different particles. Chithrani et al. (2006) and Limbach et al. (2005) directly compared the intracellular mass concentrations with particle characteristics. In line with our study, they described a dependency of particle mass uptake on the particle size with increased internal concentrations for larger particles.

The major aim of our study was the quantification of incorporated nanomaterial applying different quantification methods. We found that the three methods show similar uptake results in a concentration range of 2–8 µg Al_2_O_3_/cm^2^ cell layer. In Table 3, the advantages and disadvantages, also with regard to the applicability of the methods, are summarized, considering the sensitivity, precision, the efforts which are necessary for sample preparation and data evaluation, occurring detection limits and resolution capabilities. The acid digestion followed by neb-ICP-MS analysis provides reliable quantification results, a high sensitivity, and precision. Nevertheless, the required acid digestion has to be adjusted to the element composition of the investigated metal or metal-containing particles before the introduction of the sample to the measurement system. For certain dense metals or metal oxides, high temperatures and pressures are essential to avoid false negative results and to guarantee a homogenous sample after digestion. Furthermore, no spatial resolution within cells or distinction of dissolved and particulate metals is possible. This leads to reliable results regarding the concentration in a complete exposed cell monolayer but yields no information about the particle distribution inside individual cells and across the monolayer.

**Table 3:** Method comparison of digestion combined with nebulization-ICP-MS, LA-ICP-MS, and flow cytometry considering obtained data of this study and literature data

Parameter	neb-ICP-MS	LA-ICP-MS	Flow cytometry
Sample preparation	Washing, cell counting, adjusted acid digestion	Washing	Washing
Sensitivity	ng/l-range	mg/l-range	mg/l-range
Precision	+++	++	+
Element specificity	Yes	Yes	None
Particle size limits	None	Yes	Yes
Calibration	Solution standards	Spiked agarose gels	Particle slurries
Measurement speed	High	Low	High
Data evaluation	Established	Complex	Medium
Spatial resolution	None (homogenous sample)	Potentially high down to low µm-range (intracellular level)	High (single cells are investigated)

In addition to digestion combined with neb-ICP-MS analysis, another element-specific technique was applied in this study. The LA-ICP-MS method is already well established for the element analysis of geological and archeological samples, by the application of available standard references for calibration (Russo et al. 2002). During our experiments, the measurement system was adapted to biological samples; especially, the laser ablation of thin organic layers has been further developed. Therefore, spiked agarose gels for an external calibration approach according to earlier studies (Stärk and Wennrich 2011) were applied. The measurement of these particle spiked gels revealed a particle size effect. This effect was identified by element intensity suppression, which led to a concentration underestimation for larger particles (Fig. 4, Alu3). Such particle size limits have already been described in other studies (Thompson et al. 1990; Guillong and Günther 2002). The LA-ICP-MS method has the advantage to require no further sample preparation. It allows the measurement of a cell layer grown and exposed on glass slides. Further developments with respect to the optical resolution promise, a direct investigation of the particle internalization on the cellular level was down to a resolution of 1 µm, as already shown to operate by Drescher et al. (2012) and Wang et al. (2013). The method disadvantages refer to the instrumentation setup, the particle properties, and the data evaluation. Especially, particle size limits, transport efficiency of particles by the aerosol, or limits according to optical particle properties are important and have to be considered (Durrant 1999; Guillong and Günther 2002).

In comparison to the ICP-MS-based methods, the flow cytometry technique provides a rapid method that does not require a complex sample preparation and data evaluation. It is already implemented for the detection of nanoparticles in cells in a qualitative way, especially in the field of medicine (Suzuki et al. 2007; Zucker et al. 2010; Busch et al. 2011). The feasibility of this method for quantification of internalized nanomaterial has not yet been systematically investigated. In this study, it is shown that the results of this indirect method lay in a comparable range to direct element-specific analytical measurements. However, the method poses the disadvantage that the linear calibration curves can only be obtained up to a nominal concentration of 50 mg/l for all three particles, due to the interaction of the optical particle properties and the detection system. As the investigated particle concentrations within cells are much lower, these limitations did not influence the results of this study but have to be considered separately for each nanomaterial in future studies. The difficulty of the measurement of particle solutions by flow cytometry over a broad concentration range has already been described by Chandler et al. (2011). The irregular shape of cells, the detection limits regarding particle size and shape, and the optical properties of the scattering object (e.g., refractive index) were mentioned as critical points for the detection of nanoparticles within cells using flow cytometry (e.g., Chandler et al. (2011); Zucker et al. (2010); Stringer et al. (1995)). Additionally, Palecanda and Kobzik (2000) described the problem of a missing capability to distinguish between surface bound and incorporated particles. An advantage of flow cytometry is the potential combination with other biological investigations (e.g., fluorescent staining of specific proteins of interest or stress-related biomarkers). Additionally, cells are separated and measured individually, which offers a high resolution, in order to investigate single cells or e.g., the variability in the particle uptake behavior between cells. Furthermore, this method can be applied to non-metal-containing particles, such as carbon-based materials which are not accessible using ICP-MS-based methods. The optimization to correlate SSC values to typical distribution patterns or the agglomeration behavior of particles could be the task of future developments. In summary, particle size limitations of analytical methods using optical devices were demonstrated for LA-ICP-MS and flow cytometry. In addition, the results obtained with the semi-quantitative method of flow cytometry allow conclusions about the internalization of particles within cells and concentration-dependent trends.

## Electronic supplementary material

Below is the link to the electronic supplementary material.

Supplementary material 1 (DOCX 4803 kb)
