# Supplementary material for: Quantification of Al2O3 nanoparticles in human cell lines applying inductively coupled plasma mass spectrometry (neb-ICP-MS, LA-ICP-MS) and flow cytometry-based methods
Source: J Nanopart Res. 2014 Aug 8;16(9):2592. doi: 10.1007/s11051-014-2592-y (PMC4176630; doi:10.1007/s11051-014-2592-y)
Supplement: Supplementary file 1 — Supplementary material 1 (DOCX 4803 kb) [file 11051_2014_2592_MOESM1_ESM.docx]

**Supplementary Material**

**Quantification of Al_2_O_3_ nanoparticles in human cell lines applying inductively coupled plasma mass spectrometry (neb-ICP-MS, LA-ICP-MS) and flow cytometry based methods**

Steffi Böhme^1*^, Hans-Joachim Stärk^2^, Tobias Meißner^3^, Armin Springer^4^, Thorsten Reemtsma^2^, Dana Kühnel^1^, Wibke Busch^1^

Correspondence: Steffi Böhme, steffi.boehme@ufz.de, Helmholtz-Centre for Environmental Research - UFZ, Department of Bioanalytical Ecotoxicology, Permoserstr. 15, 04318 Leipzig (Germany)

**Particle internalization and distribution within cells**

Results

**
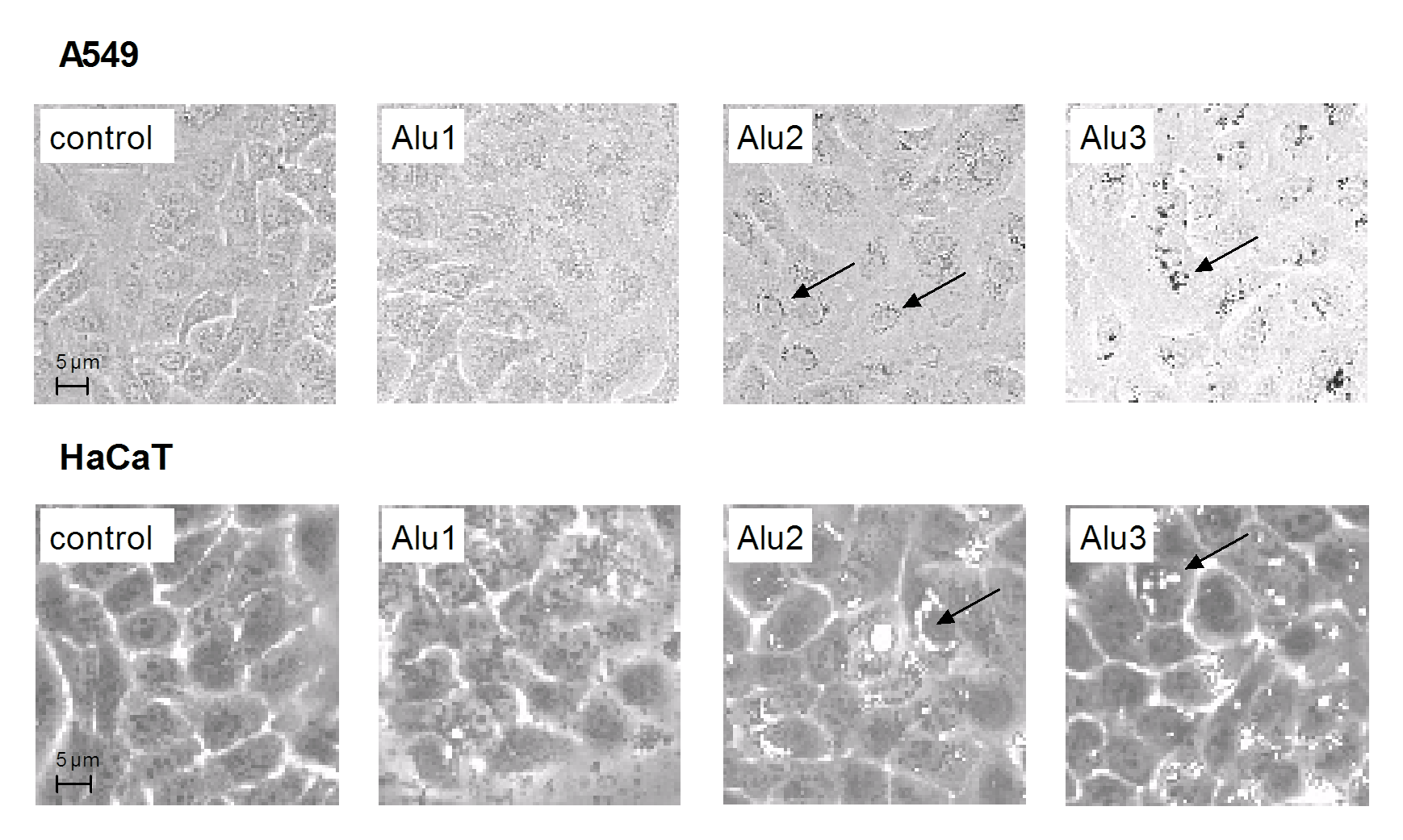
**Using light microscopy particle agglomeration and sedimentation on the cell surface was observed. Particles were found to be localized inside the cells. These observations gave first evidence that particles were internalized by both cell lines. A homogenous distribution of the smallest- (Alu1) and a heterogeneous, random localization of the micron-sized particles (Alu3) was observed (**Fig. S1**). Additionally, the formation of perinuclear rings by the middle-sized particles (Alu2) occurred.

**Fig. S1** Light microscopy images of both cell lines exposed for 24 h to the three types of aluminum oxide particles at the highest test concentration (10.4 µg Al_2_O_3_ / cm² cell layer). Light microscopy images were obtained with the transmission mode (A549) or the inverse mode (HaCaT). The formation of ring patterns (Alu2) or agglomerates (Alu3) within the cytoplasm is marked by an arrow

To prove the internalization of the particles into the cells, scanning electron microscopy coupled with an element detection unit (SEM / EDX) was performed with fixated and sectioned samples as described. The micrographs of the human keratinocytes (HaCaT) are shown in **Figure S2** in addition to the results obtained for the human lung epithelial cells (A549) that are included in the article. In contrast to the A549 cells, the internalization of the smallest particles (Alu1) in HaCaT cells could not be clearly confirmed by this method.

**
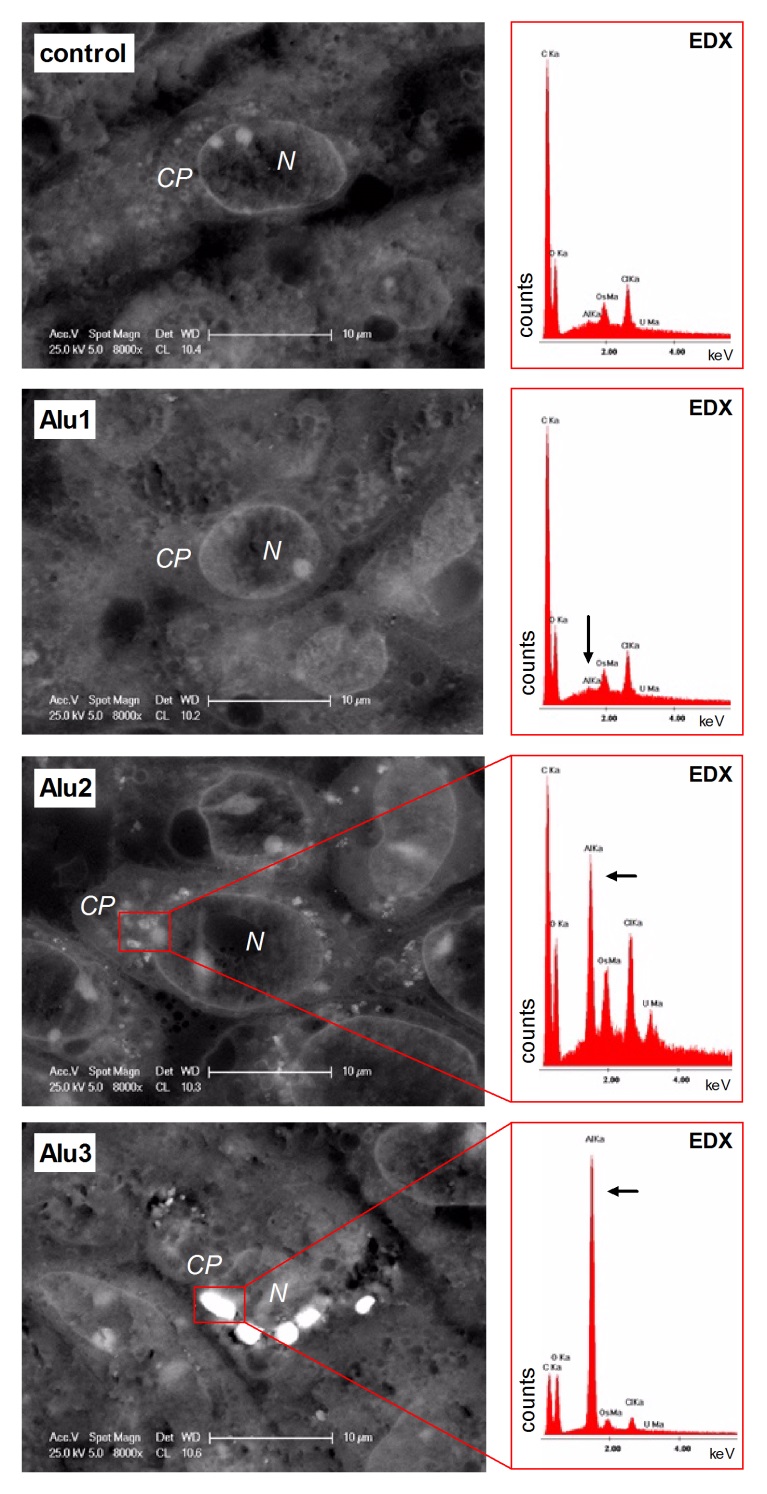
**

**Fig. S2** SEM micrographs of sections of embedded human keratinocytes (HaCaT) after 24h exposure with 10.4 µg Al_2_O_3_ / cm² cell layer and medium without particles (control). Heavy elements (e.g. aluminum) appear as light areas, which were used for EDX measurements (red rectangles). Alu2 and Alu3 particles were incorporated by the cells and were located in the cell cytoplasm (*CP*). No particles were detected in the cell nucleus (*N*)

**Cell viability assays**

Materials and methods

Potential toxic effects of aluminum oxide particles were investigated using a combined fluorescence cell viability test. Two different fluorescent indicator dyes were used: AlamarBlue (Biosource) to measure the cellular metabolic activity, and 5-carboxyfluorescein diacetate, acetoxymethyl ester (CDFA-AM, Molecular Probes, Eugene) to investigate changes in the cell membrane integrity. Assays were performed according to earlier protocols of [Schirmer et al. (1997](#_ENREF_3)), which have already been successfully applied for the toxicity testing of nanoparticles in mammalian cell lines ([Bastian et al. 2009](#_ENREF_1); [Kühnel et al. 2012](#_ENREF_2)). After the exposure with the particles for 24 h, cells were incubated for 30 min in the dark with the combined solution of both dyes (5 % (v/v) of AlamarBlue and 4 M CDFA-AM in PBS). Finally, the fluorescence was analyzed with a fluorescence plate reader (GENios Plus, Tecan). The wavelengths for excitation/emission were 530/595 nm for AlamarBlue and 493/541 nm for CFDA-AM. All results were related to 100 % viability concerning to water control.

Results

**
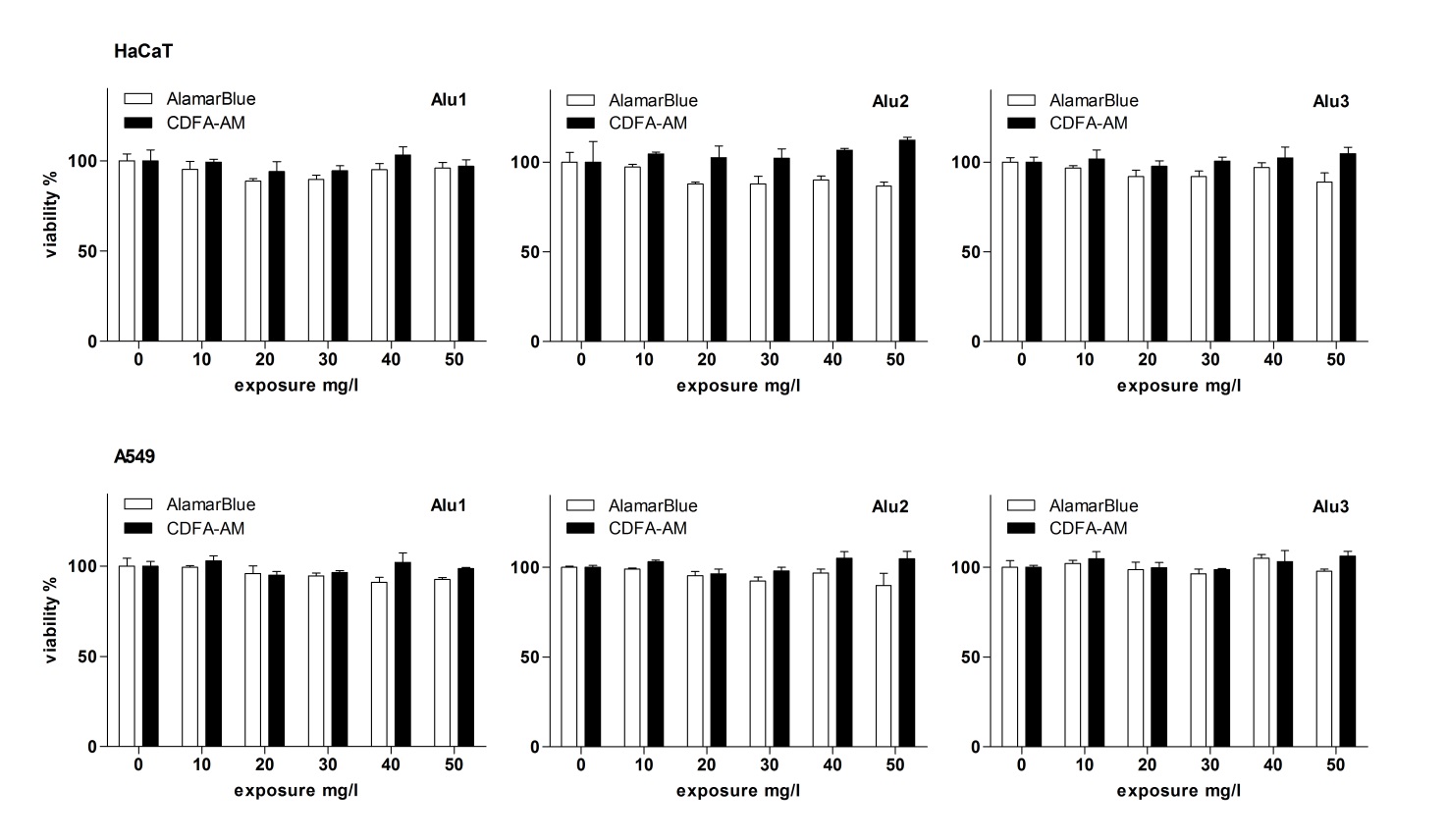
**For all types of particles and both cell lines no toxicity could be observed even at the highest test concentration of 50 mg/l, which equals 26.3 µg Al_2_O_3_ /cm² cell layer (**Fig. S3**). A slight, but not significant, decrease of the metabolic activity was observed for HaCaT cells after the exposure to the middle-sized particles (Alu2) in four of five applied concentrations.

**Fig. S3** Cell viability tests after 24 h of exposure. The results measured with the two different dyes are presented in one diagram for each particle type and each cell line. Data are shown as the mean ± standard deviation of three independent biological replicates

**Quantification of nanoparticle uptake by nebulization-ICP-MS**

Results


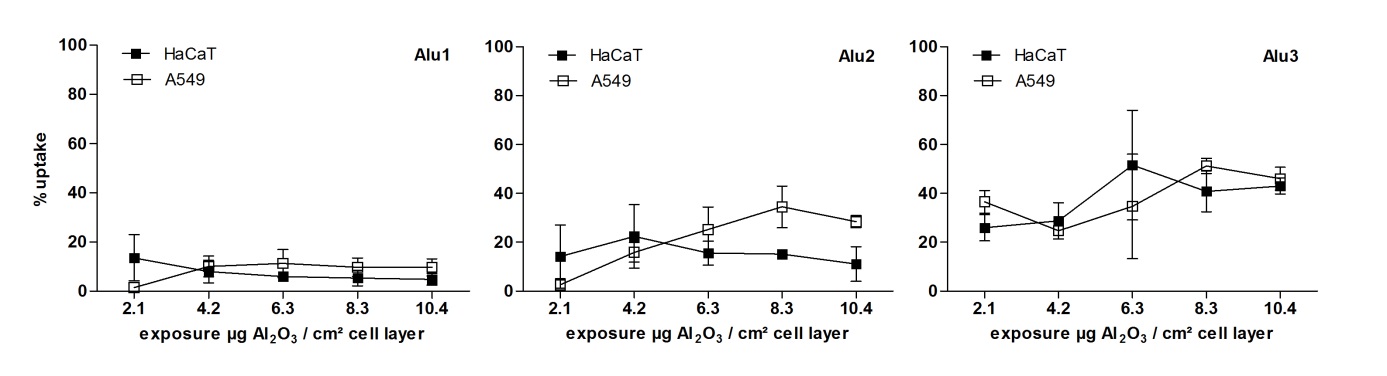
The results of the quantification of particle uptake by the cells were converted into percent of initial exposure concentrations (relative uptake rate) and are shown in **Figure S4**.

**Fig. S4** Relative uptake rates compared to initial exposure concentrations. The results are calculated by conversion of the neb-ICP-MS results. Results are presented for each particle type, both cell lines, which were exposed for 24 h, in one diagram. Data are shown as mean ± standard deviation of at least 3 independent replicates

**References**

Bastian, S., W. Busch, D. Kühnel, A. Springer, T. Meißner, R. Holke, et al. (2009). Toxicity of tungsten carbide and cobalt-doped tungsten carbide nanoparticles in mammalian cells in vitro. Environ Health Perspect 117(4): 530.

Kühnel, D., K. Scheffler, P. Wellner, T. Meißner, A. Potthoff, W. Busch, et al. (2012). Comparative evaluation of particle properties, formation of reactive oxygen species and genotoxic potential of tungsten carbide based nanoparticles in vitro. J Hazard Mater 227: 418-426.

Schirmer, K., A. Chan, B. Greenberg, D. Dixon and N. Bols (1997). Methodology for demonstrating and measuring the photocytotoxicity of fluoranthene to fish cells in culture. Toxicology in vitro 11(1): 107-119.
